# Developing the content of a locomotor disability scale for adults in Bangladesh: a qualitative study

**DOI:** 10.1186/s40945-017-0035-7

**Published:** 2017-06-06

**Authors:** Ilias Mahmud, Lynda Clarke, George B. Ploubidis

**Affiliations:** 1 James P Grant School of Public Health, BRAC University, 5th Floor, icddr, b, Mohakhali, Dhaka Bangladesh; 2 College of Public Health and Health Informatics, Qassim University, Al Bukayriah, Al Qassim Saudi Arabia; 3 Department of Population Health, London School of Hygiene & Tropical Medicine, Keppel Street, London, WC1E 7HT UK; 4 Department of Social Science, Centre for Longitudinal Studies, University College London, 55-59 Gordon Square, London, WC1H 0NU UK

**Keywords:** Disability, Locomotor disability, Physical disability, Locomotor disability scale, Bangladesh

## Abstract

**Background:**

Bangladesh has an estimated 17 million adults with disabilities. A significant proportion of them are believed to have locomotor disabilities. There are over 300 non-governmental organizations providing different types of rehabilitation services to them. However, there is no locally developed and validated locomotor disability measurement scale in Bangladesh. The purpose of this study was to develop a locomotor disability scale with disability indicators suitable for adults in Bangladesh.

**Methods:**

Semi-structured interviews were conducted with 25 purposively selected adults with locomotor disabilities to generate scale items. At the second stage, cognitive interviews were conducted with 12 purposively selected adults with locomotor disabilities in order to refine the measurement questions and response categories. Data were analysed using the framework technique- identifying, abstracting, charting and matching themes across the interviews.

**Results:**

For a locomotor disability scale, 70 activities (disability indicators) were identified: 37 mobility activities, 9 activities of daily living, 17 work/productivity activities and 7 leisure activities. Cognitive interviews revealed that when asking the respondents to rate their difficulty in performing the activities, instead of just mentioning the activity name, such as taking a bath or shower, a detailed description of the activity and response options were necessary to ensure consistent interpretation of the disability indicators and response options across all respondents.

**Conclusions:**

Identifying suitable disability indicators was the first step in developing a locomotor disability scale for adults in Bangladesh. Interviewing adults with locomotor disabilities in Bangladesh ensured that the locomotor disability scale is of relevance to them and consequently it has excellent content validity. Further research is needed to evaluate the psychometric properties of this scale.

**Electronic supplementary material:**

The online version of this article (doi:10.1186/s40945-017-0035-7) contains supplementary material, which is available to authorized users.

## Background

Disability is an umbrella term of impairments, activity limitations and participation restrictions [1]. The World Health Survey 2002–2004 estimated that 18% of adults aged 18 years and over in low-income countries experience disabilities [2]. Based on this estimate Bangladesh, a country with a population over 152 million [3] of whom 61.6% is aged between 15 and 64 years [4], is estimated to have approximately 17 million adults with disabilities. The proportion of adults with disabilities which have mobility impairments is uncertain, but the number is undoubtedly high in Bangladesh. Mobility impairments are defined as significant problems in body function and structure which result in difficulty in mobility, such as paralysed legs. Individuals’ mobility impairments in association with their contextual factors (personal, social, political and physical environmental features) may limit their activities and restrict their participation in real life situations, a phenomenon termed as ‘locomotor disability’ (LD) in this article.

There are over 300 international, national and local non-government organizations (NGOs) actively providing services to persons with disabilities (PWDs) in Bangladesh [5]. Many of these NGOs provide rehabilitation services to people with locomotor disabilities (PLDs). However, these organisations lack a locally developed, or adapted, reliable and valid scale to assess severity of LD and evaluate the LD rehabilitation outcome in PLDs. Disability measurement scales developed and validated elsewhere might lack relevance to people in Bangladesh since severity of disability does not only depend on impairment but also the contextual factors [1]. These contextual factors, such as physical environment, culture, attitudes of the community towards disability and PWDs may vary country to country. On the other hand, to successfully evaluate a disability rehabilitation programme, a measure of disability which is not limited to use in institutional settings, but can also be used in the community is required to assess how a PWDs is coping with everyday life. Some existing disability measurement scales, developed elsewhere, to assess functional performance, such as the Functional Independence Measure (FIM), and the Barthel Index (BI), were primarily developed for inpatient rehabilitation [6, 7], and thus might not be suitable for evaluating functional performance in the community.

Some other instruments, developed outside of Bangladesh, were designed using a medical model of disability and focus only on individuals’ mobility to measure disability level but ignore performance in activities of daily living (ADL)/self-care, work/productivity and leisure activities [6, 8–12]. However, measuring abilities in mobility might not adequately predict disability [11], since disability implies a complex and dynamic interaction between individual health conditions and contextual factors, such as the environmental and personal factors [1].

Some disability measurement scales, such as the High Level Mobility Assessment Tool (HiMAT) [6] and the World Health Organization Disability Assessment Schedule 2.0 (WHODAS 2.0) [13, 14], have been developed primarily by reviewing existing literature and consulting experts. Not involving the target population in scale development might result in a difference in understanding the meaning of the questions and response categories between the respondents and researchers [15]. The resultant measurement errors might increase the likelihood of making wrong inferences about rehabilitation outcomes [16].

The aim of this study was to contribute to the knowledge base relating to the measurement of disabilities experienced by adults with mobility impairments in Bangladesh by developing an instrument defined by individuals with such impairments. The specific objective was to identify disability indicators (items) suitable for a locomotor disability scale (LDS) for adults in Bangladesh. Purpose of this instrument is to assess LD among adults in the community and evaluate rehabilitation outcome in adults with LDs in simulated or real community settings.

## Methods

### Research design

Semi-structured interviews of adults with LDs were conducted in order to generate disability indicators [17, 18]. In the second phase, cognitive interviews were conducted to understand whether respondents endorse the disability indicators identified through previously conducted semi-structured interviews and whether they understand the indicators and response options of the newly developed LDS [18, 19].

### Sampling

Twenty five adults with LDs, 15 males, 10 females, 13 rural residents, 12 urban residents, were sampled purposively, ensuring representation from different socio-demographic and disability groups, for the semi-structured interviews from the adults with LDs accessing out-patient rehabilitation services at the Centre for the Rehabilitation of the Paralysed (CRP). In addition, a different set of twelve adults with LDs, six males and six females, six rural and six urban residents, were selected purposively for the cognitive interviews.

The CRP is a NGO located in Dhaka, Bangladesh. It provides both institutional-based and community-based rehabilitation services to PLDs, managed by a multidisciplinary team involving medical doctors, nurses, physiotherapists, occupational therapists, speech and language therapists, trained staff in orthotics and prosthetics, counsellors and social workers [20].

Respondents’ academic qualifications ranged from none to 16 years of formal education. They had a range of diagnoses, such as spinal cord injury, stroke, polio, and other neuromuscular or musculoskeletal conditions. Respondents were considered eligible for this study if they were living in a community setting for at least over the past 1 month with their disability following acute medical care and were aged between 18 and 65 years. Persons with cognitive and psychiatric co-morbidities, and those who were unable to speak were excluded from the study. Persons with any medical emergencies were also excluded.

### Data collection

All semi-structured and cognitive interviews were conducted by two trained interviewers. The semi-structured interviews were organised around a set of pre-defined open ended questions, shown in Additional file 1, while further questions emerged from the dialogues between the interviewers and respondents. The topic guide of the semi-structured interviews was prepared by reviewing the literature and consulting experts to elicit respondents’ disability experiences in mobility and every domain of occupational performance: ADL/self-care, productivity/work and leisure activities.

For the cognitive interviews, respondents were first asked the questions and requested to select a response options prior to discussing the questions. A flash card with response options was displayed in front of the respondents throughout the interviews. Later, respondents were requested to give feedback on their understanding of the questions and associated response options, and to verbalise the reasoning they had used in producing their answers. Cognitive interviews were conducted in two rounds. Following the first round of six interviews questionnaire was revised, and a second round of six interviews was conducted to ensure that the problems identified through earlier interviews had been rectified and that no new problems had arisen in the revised version. The first and second round sample cognitive interview questions are presented in Additional file 1. Unlike the semi-structured interviews, the cognitive interviews were not recorded; instead, the interviewers took notes of their responses and any problems identified.

### Data analysis

In the first phase, semi-structured interviews were analysed and 70 items for the scale were identified. Immediately after the interviews verbatim written transcripts were prepared from the digitally recorded interviews. The researcher read through each interview transcript several times to identify the problem statements and the domains of problems. In this regard, the framework technique [21] was employed. This technique involved identifying, abstracting, charting and matching themes (problem statements and domains) across the interviews. All items/activities identified were considered for the scale, but statements were consolidated where it was perceived that the underlying difficulties were responsible for causing problems in the same activity. For example, statements which described problems with feeding because of poor sitting balance, or because of difficulty in collecting cooked/ready to eat food items, were grouped together under the domain ‘feeding’. The domains of problems were later grouped under four broad categories: mobility, ADL/self-care activities, work/productivity and leisure activities.

Following analysis of semi-structured interviews, a preliminary questionnaire was prepared and cognitive interviews were conducted. Cognitive interview analyses was focused on identifying dominant trends (problems identified repeatedly) across interviews. Problems that had been identified through a single interview, but nonetheless had the potential to cause serious problems later in data collection, or which might be reasonably common in the target population were also noted (for example, difficulties in interpreting an item or associated response options) [22].

## Results

### Developing the contents

Problem statements generated through the analysis of semi-structured interviews were related to 70 domains (activities) which were included in the Locomotor Disability Scale (LDS) as disability indicators (items). Problem statements related to individuals’ mobility were summarised under the umbrella of 37 mobility activities. These are presented in Table 1. Problem statements related to occupational performance areas were summarised under 33 activities. These 33 activities were further categorised as ADL, work/productivity and leisure activities (Table 2). The complete LDS, and how the total score should be calculated and missing values should be handled are presented as an Additional file 2.

**Table 1:** List of mobility activity items

Mobility activities		
1. Sitting up from lying down	14. Maintaining a squatting position	27. Walking in quick steps
2. Lying down from sitting up	15. Maintaining a sitting position on a chair	28. Jumping
3. Standing up from sitting on a chair	16. Maintaining a sitting position on the floor	29. Moving inside the home using a wheelchair
4. Sitting down on a chair	17. Maintaining a standing position	30. Moving in the neighbourhood using a wheelchair
5. Sitting down on the floor	18. Walking inside the home	31. Carrying objects
6. Standing up/sitting up on a wheelchair from a sitting position on floor	19. Walking in the neighbourhood	32. Getting into and out of own home
7. Getting into a squatting position	20. Walking on different surfaces	33. Travelling by non-motorised vehicles
8. Getting out of a squatting position	21. Walking around obstacles	34. Travelling by private motorised vehicles
9. Bending down and sideways	22. Crawling inside the home	35. Travelling by public transports
10. Lifting objects	23. Crawling in the neighbourhood	36. Driving non-motorised vehicles
11. Reaching for overhead objects	24. Rolling inside the home	37. Driving motorised vehicles
12. Transferring oneself from a sitting position to another sitting position	25. Rolling in the neighbourhood	
13. Maintaining a lying position	26. Climbing up and down two flights of stair

**Table 2:** List of the ADL, work/productivity and leisure activity items

Activities of daily living	Work/productivity activities
1. Washing parts of body	17. Shopping
2. Taking a bath or shower	18. Cooking
3. Grooming	19. Serving meals
4. Toileting	20. Cleaning cooking area and utensils
5. Dressing	21. Washing and drying clothes and garments
6. Feeding	22. Cleaning living area
7. Maintaining own health	23. Disposing of household garbage
8. Praying	24. Maintaining dwelling and furnishings
9. Attending ceremonies	25. Taking care of domestic/pet animals
Leisure activities	26. Assisting household members with self-care
10. Socialising	27. Assisting household members in movement
11. Playing in-door games	28. Assisting household members in health maintenance
12. Playing out-door games	29. Accessing public services
13. Attending arts, cultural and sports events	30. Seeking employment
14. Travelling for pleasure	31. Maintaining an occupation
15. Gardening	32. Agricultural activities
16. Watching television	33. Doing voluntary social work

### Evaluating and refining the contents

Cognitive interview respondents expressed the view that no relevant activities were missing in the LDS. The first round cognitive interview respondents demonstrated some difficulties and inconsistencies in comprehending double questions. In addition, they inconsistently interpreted the LD indicators and response options when those were not clearly defined. However, the second round cognitive interview respondents demonstrated that they understood and interpreted the LD indicators and response options as intended.

#### Double questions

Some respondents found it challenging to reply to any double questions. For example, when asked about their difficulties in ‘getting into and out of a seated position on a chair’, one respondent replied: *“[I] don’t have any problem in getting into a seated position [on a chair], but in order to stand up from a seated position [on a chair] I need to grab the wall or something else.”* Hence, after the first round of cognitive interviews these questions were broken down into two parts, such as ‘standing up from sitting on a chair’ and ‘sitting down on a chair’. In few cases, where double questions were retained for brevity, respondents were asked to rate the part most difficult for them, such as ‘getting into and out of own home’.

#### Vague disability indicators

There was evidence from cognitive interviews that LD indicators which did not have a definition were sometimes interpreted differently by different respondents. For example, when the respondents were asked about feeding difficulties different respondents interpreted ‘feeding’ differently. One assumed that food would be served, so replied: “*I don’t have any difficulties. My legs are paralysed, not my hands. I can eat without any difficulties*”. While another respondent, with similar impairment, assumed that feeding activity also involves collecting food items. He replied: *“I experience huge difficulty in feeding. One day my mother was not at home during lunch time, so I was not fed. I could not bring food items from kitchen”.*

LD indicators without a definition not only resulted in variable understanding between respondents but also inconsistencies in interpreting different indicators by the same respondent. For example, a first round cognitive interview respondent rated his difficulties in ‘washing body parts’ as moderate. He said: *“My [standing] balance is poor. It is difficult to lift water [from the water container] while standing, that is there is moderate difficulty in lifting water with a jug.”* However, the same individual rated his difficulty in bathing as ‘mild’. He justified this by saying: “*I can lift water with a jug [from the water container] while sitting on a chair, hence can perform bathing without much difficulty.”* In this scenario, a family member might assist him by putting a chair in his bathroom before he takes bath but not when he washes his body parts. It was clear that this individual was dependent on others for his bathing; therefore, it was not correct that his difficulties in bathing were rated as ‘mild’ according to the scale we used (see Table 3).

**Table 3:** Response options for the locomotor disability scale

Difficulty levels	Score
No difficulty	0
Can perform on your own without any difficulties or negligible difficulties.	
Mild difficulty	1
Can perform on your own but with mild difficulties, such as requires more than usual time and effort. No risk involved and does not require any assistance of others.	
Moderate difficulty	2
Cannot perform on your own; requires mild physical assistance or supervision of one person. If perform alone then there is a risk/history of fall or a long time period and/or severe effort is required.	
Severe difficulty	3
Cannot perform on your own; need moderate physical assistance of one person.	
Extreme difficulty	4
Cannot perform on your own. Need total or maximum physical assistance of others (ideally two persons).	
Not applicable	99
Do not or cannot perform because of reasons not related to disability.	

Therefore, uniform definitions for each of the 70 LD indicators were proposed and it was recommended that the interviewers read these definitions exactly as they were printed to the respondents when conducting interviews. The uniform definitions for the 70 disability indicators of the proposed LDS were prepared by reviewing the 25 in-depth and 12 cognitive interviews, and also by reviewing the existing literature, particularly the International Classification of Functioning, Disability and Health (ICF) [1]. For example, the question assessing difficulty to ‘take a bath or shower’ asks: *currently, how much difficulty do you have in ‘taking a bath or shower’? ‘Taking a bath or shower’ includes accessing the designated place and obtaining and applying water, soap and other substances to the whole body in order to clean oneself and then drying whole body using a towel or other means.*

#### Unfamiliar language and terms

Selecting language and terminology which are understood by the respondents is important in order to ensure that they interpret the questions as intended. For example, when asked about difficulties in lifting objects, one respondent replied “[I] need assistance to lift a heavy object”. Consequently, in the LDS, the question related to lifting an object asks: currently, how much difficulty do you have in lifting any object weighing approximately two kilograms, such as a jug full of water or other everyday objects?

Another respondent with limited education (did not complete secondary schooling) did not understand the Bangla term for ‘toileting’, ‘*shouchakarj*a’. When asked about his difficulties in *‘shouchakarja’* he replied: *“this activity is not applicable for me*”. When he was asked about his understanding about this activity, he replied: *“by this activity I understand shouchakarja”.* Clearly, he did not understand the meaning of ‘*shouchakarja’*, as he said ‘*shouchakarja’* (toileting) was not relevant to him. Hence, the LDS used common language instead of just standard book language.

#### Vague response options

Cognitive interviews also enabled the development of clear response options with clear definitions: no difficulty, mild difficulty, moderate difficulty, severe difficulty and extreme difficulty. These response options were adapted from the ICF [1]. Analysis of the first round cognitive interviews revealed that respondents interpreted these difficulty levels differently. For example, when asked about their difficulties in walking inside the home, one person with arthritis and another with stroke rated their difficulties as severe*.* When they were asked about the reasoning for that rating, the person with arthritis replied: “*I feel pain in my ankle while walking”,* while the person with a stroke said: *“I can’t walk alone. I need somebody to hold me while walking”.* In this example, one person could walk, though with pain, while another was unable to walk without physical assistance from another individual. Such disparities in interpretation of difficulty levels between respondents prompted the development of more clear and distinguishable response options which are presented in Table 3.

## Discussion

This paper discusses the development of the LDS, an instrument to measure locomotor disabilities (LDs) in adults. This scale was developed in accordance with the methods [15, 18, 19] required to ensure the content validity, the relevance of the disability indicators to adults in Bangladesh. To ensure that respondents interpreted the measurement questions and response options as intended, disability indicators for the LDS were developed though semi-structured and cognitive interviews with adults with mobility impairments. Interviewing adults with LDs gave us a greater insight into the disabilities they were experiencing and enabled us to explore the issues most important to them, which helped to avoid making assumptions or imposing purely academic perspectives on them. Also, interviewing them allowed a better insight into the language and terminologies they used to express their disabilities, and thus, ensured that the language used in the LDS was understood by the target population (adults in Bangladesh) [18, 23].

The qualitative evidence showed that mobility impairments affect individuals’ mobility, and consequently their participation in ADL, work and leisure activities.

### Mobility activity items

The mobility problems identified by the respondents and therefore included in the LDS are consistent with the ICF mobility items [1] and includes all mobility related items of other commonly used disability measures, such as the WHODAS 2.0 [13], the BI [24], the FIM [25] and the Rehabilitation Activities Profile (RAP) [7].

The ICF item ‘sitting’ involves both getting into a sitting position with bent legs or crossed legs and getting out of that sitting position [1]. In the western world, the majority of people usually sit on a chair, while in Bangladesh sitting on the floor or a very low seat are widely practiced. Women in Bangladesh are traditionally responsible for cooking and they perform all or most of their cooking tasks at floor level [26]. Getting into and out of a sitting position at floor level requires more muscle activities than getting into and out of a seated position on a chair or other seats of similar height. Thus, in addition to ‘sitting down on a chair’, the results of the semi-structured interviews suggested the addition of ‘sitting down on the floor’. In the ICF, lying down, sitting, squatting and standing are used for both getting into and out of that position [1]. But, moving against gravity requires more muscle strength than moving towards gravity [27], in addition, our cognitive interview respondents experienced difficulties in replying double questions. Thus, measuring getting into and out of these positions separately, as suggested by this scale, is likely to be more reliable.

Getting into and out of a squatting position, and maintaining this position is important in the Bangladeshi context. In Bangladesh, women usually cook at floor level, which necessitates repeatedly get into and out of a squatting position as well as maintaining it. In addition, in Bangladesh people usually do not use a high toilet but an Asian type toilet (low level squatting toilet) and consequently it is important that people are able to get into and out of a squatting position and maintain it.

Our qualitative data suggested replacing the ICF items ‘walking short distance (less than a kilometre)’ and ‘walking long distance (more than a kilometre)’ [1] by ‘walking inside the home’ and ‘walking in the neighbourhood’ respectively. This is also a contrast to the WHODAS 2.0 which include ‘walking a long distance such as a kilometre or equivalent’ [13]. This was found to be preferential as people tend not to be able to estimate the distance they walk and hence find it difficult to answer these types of questions [15], but can answer whether they could walk inside or outside of their home. This is important as a disability measure should not measure an individual’s theoretical ‘capability’ but ‘performance’ in real life situations. Additionally, measuring the difficulty in walking inside and outside the home, rather than relying to the length of distance walked, captures the role of environmental factors in disability.

Walking outside the home or in the community can be a big challenge for people with LDs because of difficulty in access (inaccessibility). In Bangladesh, the needs of the pedestrians are not properly considered when roads are built. Often there are no footpaths at all or, if present, these are very narrow and thus always overcrowded. They often stop suddenly, have uneven surface and potholes, are obstructed by signboards, garbage, dustbins, illegal street vendors, and illegal parking and illegal driving [28]. Thus walking on the roads is difficult even for non-disabled people.

We found that even people with mild mobility impairment often experience severe difficulties in walking and moving outside the home. The inaccessible built and natural environment and negative attitudes of others towards disability and PWDs perhaps play a role [26, 29], since disability does not only result from an individual’s impairment but its interaction with environmental and personal factors [1]. These findings further necessitate the importance of a locally developed disability measurement scale which can incorporate more salient local issues, such as squatting for cooking and toileting.

Respondents also mentioned difficulties or inabilities in travelling from one place to another. The common motor vehicles in Bangladesh are motorcycles, car, auto rickshaw, bus, train and launch (a boat which has an engine and carries passengers). However, the majority of the transport demand is met by non-motorised (human powered) rickshaws [30]. All of these vehicles are inaccessible for users of mobility aids such as a wheelchair, walking frame or crutches [26, 31]. Furthermore, the number of vehicles operating on the roads is in excess of the roads’ actual capacity, but is still inadequate to meet the demand [30]. These vehicles are always overcrowded and are difficult even for non-disabled people to use and it is almost impossible for PWDs and/or their caregivers to carry mobility aids like a wheelchair, walking frame or crutches while travelling on overcrowded and inaccessible public transport [31], as reported by many respondents including Shajna, a 19 year old girl with spinal cord injury:

*“In case of travelling by bus, somebody needs to carry me on and off the bus on his/her lap. Furthermore, it would take some time, so the bus would have to wait for me, but it would not because I am not a VIP [very important person]. The drivers get angry and talk bad. That is why, I do not go anywhere. Moreover, I need to carry my wheelchair with me, but this is almost impossible to do on any bus.”*

While Shajna was unable to walk and needed a wheelchair to move, those who can walk with some difficulties also find it impossible to travel by public bus [26], such as Akhi who could walk with crutches said:

*“I cannot get on a local bus [public bus] because the bus stops only for 1 minute, but I need time to move. Say, I need 4–5 min even just to reach to the door of the bus, in that case, the bus leaves without me. On the other hand, these buses are very crowded. Always 4-5 people try and push each other to get on the bus through the door at a time. I would have to do the same if I want to travel by bus, but with my crutches I won’t be able to do that. People would have to leave me space if I want to get on a bus, so they would be annoyed. Moreover, I would face similar problems in getting down from the bus.”*

Respondents also reported facing problems with accessibility in their own home [26]. Getting in and out of their home is a challenge. In Bangladesh, rural homes are raised from the ground level to avoid flood and rain water. In cities, people live in multi-storeyed flats, many of which do not have any lift, so inhabitants need to encounter high steps or stairs to get in and out of their home. This was mentioned by one of our wheelchair user respondents:

*“I stay either outside [in the yard] or inside of my house most of the time in any given day. I cannot get in and out of my house [alone]. Sometimes they [family members] put me outside in the morning and bring me back inside at night when I go to sleep.”*

These mobility problems ultimately hinder their participation in occupational performance areas: activities of daily living, work and leisure activities.

### Activities of daily living (ADL) items

The ADL items included in the LDS are washing parts of body, taking a bath or shower, grooming, toileting, dressing, feeding, maintaining own health, praying and attending ceremonies. These include all of the five ADL items (feeding, bathing, grooming, dressing, and toilet use) of the BI [32], and all of the self-care activities in the RAP except sleeping and maintaining continence [7]. The LDS also includes all self-care activity items of the WHODAS 2.0 except ‘staying by yourself for a few days’ [14]. However, in order to be able to live alone for a few days individuals would have to be able to perform the ADL activities included in the LDS without any assistance from others. Therefore, ‘staying by yourself for a few days’ is perhaps a redundant item for a LDS. Respondents of this study did not mention sleeping as a problem possibly because none had any acute medical condition or they might not have considered sleeping as an activity. However, problems in sleeping might have partially covered by the mobility item, ‘maintaining lying position’. Maintaining continence is not also included in the LDS like the BI [32] and the RAP [7]. Incontinence is considered as an impairment which might restrict participation in other activities, such as attending ceremonies and praying.

Unlike other measures of disabilities such as FIM [33], BI [32], RAP [7] or WHODAS 2.0 [13], it was decided to include culturally appropriate activity ‘praying’ as an item in the LDS. Irrespective of religion, education and socioeconomic background PWDs in Bangladesh seek spiritual healing for their disabling condition [34], hence, ‘praying’ is a very important activity for them.

Home modifications improve independence in ADL at home, but if the whole community is not modified or built considering the needs of PWDs then they will be confined to their own home [26]. This was reported by Siraj, a 40 year old man with a neurological condition who had problems in sitting without a seat thus had problem in using an Asian type toilet. Siraj installed a high toilet (western type) at his home and could perform toileting independently but, he was avoiding attending any social events and ceremonies because those places usually do not have a high toilet. Thus, Siraj might experience independence in toileting at home but not in outside social activities, such as ‘attending ceremonies’.

None of the respondents mentioned problems with sexual activities and consequently this was not included in the LDS. Perhaps in such a conservative society as Bangladesh, the participants felt embarrassed to talk about their sexual desires and problems in performing such activities. Also, because of the overwhelming problems inherent in living with a disability, the community, family and PWDs themselves might not give priority to sexual needs.

### Work activity items

Work or productive activity items include household management activities that the American Occupational Therapy Association (AOTA) terms as the instrumental activities of daily living [35], activities needed for paid or unpaid employment and activities needed for learning through formal and informal education [36]. The work activity items included in the LDS conform with the ICF [1], the occupational therapy practice frame work of the AOTA [35] and the Canadian Occupational Therapy Performance Measure [36], the WHODAS 2.0 [14] and the RAP [7]. While the two popular measures of disability, the FIM and BI, don’t assess performance in work activities [25, 37].

An inaccessible built environment, the negative attitudes of others and the absence or lack of enforcement of disability friendly systems, for example preferential seating on public transport for PWDs, were identified as barriers to participating in work/productive activities for people with mobility impairments. For example, Paru, a 22 year old woman who was using crutches for walking had to stop shopping as a result of inaccessible roads, transport, and markets. She said:

*“I cannot go to market for shopping because I cannot climb stairs. Furthermore, there are also problems on the roads. When there are potholes on the footpath and/or when there are stagnated water and mud I am in trouble. In the past, I used to jump these barriers, but now I cannot jump because I use crutches. Again, it is difficult for me to take the opposite footpath to avoid these barriers because there are cars on the road. Another problem is markets are very crowded. When I was well, I could make my way through the crowed. Now, since I use crutches, I need more space to walk. It is difficult to find such amount of space in a crowded place. Moreover, in a crowded place it is common to unintentionally be pushed by others, but now I won’t be able to keep my balance and would fall down.”*

It was found that Shajna, a 19 year old girl, could not even apply for her national identity card because of an inaccessible route from her home to the makeshift office of the election commission. She said:

*“I could not apply for voter identity card [national identity card] because I could not visit the camp which was three kilometres far [from our house]. There was nobody in my house who could take me there. I am adult, if somebody carries me on his/her lap, I feel ashamed. It is not also possible to travel there using my wheelchair. If I travel by van [rickshaw van] my father would have to take the trouble of carrying me to a van and from the van to a chair in that office. But I don’t not know how would they behave, how long would I have to wait; it would be difficult for me to sit there for a long time.”*

While Shajna did not apply for her national identity card for fear of being ignored by staff members, Saidul, an ex-school teacher, had experienced this attitude while visiting a government office to draw his pension. He said:

*“In the past, when I visited the local government office, they used to respect me a lot. But, now the way they behave, I don’t enjoy. In the past, when I went there for any work, the office staff used to complete my work quickly, but now they keep me waiting for a long time. Last time, I had to go there three days to draw my pension!”*

PWDs do not always encounter negative attitudes, but sometimes they experience non-constructive help. The same ex school teacher reported that he managed to continue his school job for some time because of the ‘helpful’ attitudes of his colleagues and students. The school did not ask him to take any classes, he just went to school once a week to sign for his whole week attendance and meet his colleagues and students for an hour. But, soon he felt worthless as he was not doing anything there, and his son had to accompany him to school. He had to travel by boat which was difficult. The school’s toilet (Asian type) was not accessible for him and the class rooms were also inaccessible, so Saidul decided to take early retirement.

It was found that even those who showed courage in overcoming physical (architectural) barriers faced the ‘disability-unfriendly’ systems which restricted their participation in many activities, including accessing public services for various needs. This fact was illustrated by Siddik, a young man who had poliomyelitis:

*“When I go to bank to draw money or pay electricity bill, I have to stand in a queue. I can’t remain standing for a long time, so often I have to leave the queue to sit on a chair, but in the meantime more people join the queue, and I remain seated unnoticed. I have to wait a long time to get the service. It would have been better if the bank would have a system for disabled people.”*

Saidul could not even continue the relatively easy job of teaching at a school, so more physically laborious jobs are obviously impossible for persons with moderate or severe mobility impairments. Mukhles was a 63 year old farmer in a rural area of Bangladesh. He has stopped performing agricultural activities after suffering from stroke because of his mobility problems:

*“In this condition how would I do [agricultural activities]? Suppose, if I want to do these activities I would have to go to the field and many other places. What if I fall down somewhere on mud during any rainy day? If I fall down on the mud while walking, I won’t be able to stand up alone. I might even die falling down into mud and water. Furthermore, now I cannot control my cattle. I need strength in my hands and legs to hold the rope which tied a cow. If it tries to run away I would fall down, if I want to stop it holding the rope it would drag me away.”*

### Leisure activity items

Leisure activities are intrinsically motivated, non-obligatory activities that people perform when they are not engaged in obligatory activities such as work, ADL or sleep [35, 38, 39]. Participation in leisure activities promotes better quality of life and inclusion in the community [38, 40]. However, leisure activities are often overlooked by commonly used disability measures. For example, the WHODAS 2.0 and RAP included leisure activities as a single generic item [14, 37], while the FIM and BI did not include any leisure activity [25, 37]. Respondents in this study identified very few leisure activities which could be categorised as socialising, playing in-door games, playing out-door games, attending arts, cultural and sports events, travelling for pleasure, gardening and watching television programmes. Reporting fewer leisure activity items by the respondents perhaps illustrates the fact that respondents prioritised ADL and work/productive activities much higher than non-obligatory leisure activities. Jahir, a 36 year old man with paraplegia (due to spinal cord injury) burst out laughing when the interviewer asked him about his leisure activities. This was also reported by a study with people with brain injury which found that participation in leisure activities, particularly in social leisure activities, such as socialising and participating in sports events, declines during rehabilitation compared to pre-disability levels [41].

Participation in leisure activities is largely determined by personal factors and perceived barriers rather than by disability-related factors [40]. It was found that intrapersonal reasons related to dissatisfaction with their own body, and impairments contributed to perceived restrictions in participating in different activities including socialising. For example, Siddik (quoted previously) said:

*“I feel depressed when I attend any party and see other non-disabled people wear nice shoes and walk confidently. I am not able to wear such nice shoes even though I have money to buy that. They walk so nicely and confidently, but I can’t. God made me like this, I am not sad, but sometimes I can’t hold my emotion.”*

Another respondent, 62 year old Saidul who had stroke and became hemiplegic explained the reasons for not participating in any leisure activities:

*“Peacock loves dancing and to fan its colourful tails, but when it gazes at its legs it stops dancing because its legs are ugly. My condition is same; when I look at my legs I feel depressed and lose interest from everything.”*

### Cognitive evaluation of items and response options

Analysis of the cognitive interviews suggested the need for a comprehensive definition of the listed activities in order to ensure that they are interpreted consistently and as intended by the respondents. Other measures of disability, such as the BI, WHODAS 2.0 and RAP did not include definition of activities in their measurement questions [7, 14, 37], which makes them prone to differential interpretation by the respondents. While completing these scales somebody might assume that all materials to perform the listed activities would be in place, so an individual would just have to perform them. Perhaps this might be the case for high-income countries and hospital settings, but not in community settings of low-income countries like Bangladesh. For example, in Bangladeshi villages bathrooms/toilets often do not have tap water so people need to carry water from their pond or tube-well. Thus, in the LDS the definition of the listed ADL, work and leisure activities included the tasks of obtaining necessary supplies when required for that activity, and putting those back in the usual place after performing that activity. People experience disability in a real life situation not in a standardised environment such as in a hospital setting. For example, Bindu, a female wheelchair user respondent said:

*“When my mother is not at home or when she is sick, I am not fed. At home we do not have anybody else who can cook food. Moreover, I cannot bring food from the kitchen to eat. My mother serves food for me and then I eat. So, when she cannot do that I starve”.*

This lady would have rated as independent in feeding in the BI [42] but not in the LDS. In Bangladeshi villages the kitchen is usually detached from the house and both are raised from the ground to avoid flood/rain water thus have steps at entrance. Incorporating real life situations in the definition of the activities included in the LDS improved the validity of measurement of disabilities. This has also implications for rehabilitation programmes as they should focus on maximising independence in community settings not in any standardised hospital environment.

It was also observed through the cognitive interviews that interpretation of the response options varied between respondents and often was not interpreted as they were intended. Consequently, the LDS included clear definition of the response options and hence optimised the consistency in interpretation between the respondents and ensured that these were interpreted as intended. Cognitive interviews were also instrumental in avoiding double questions, vague questions and unfamiliar terminologies. A qualitative evaluation of the Short-Form 36 Health Status questionnaire (SF-36) found that respondents found it difficult to answer these types of questions [15] and so did the cognitive interview respondents of this study. Another qualitative evaluation of the WHODAS 2.0 in a community setting in the UK reported that unambiguous wording of this instrument cause uncertainty among respondents in relation to contextual aspects of disability [43].

Disability does not only result from an individual’s physical impairment but it’s interaction with the contextual factors [44]. Therefore, it is important that disability measurement scale is sensitive to any change in contextual factors (for example, introduction of a facilitator such as assistive device or a barrier such as unavailability of assistive device). Addition of culturally appropriate activity items and culturally appropriate definitions of these activity items, and asking respondents to rate their levels of difficulties in performing activities even after using their usual assistive devices is expected to enable our scale to be sensitive to any change in the contextual environment.

There were some limitations in the methods of this study. All interviews were conducted at CRP. People who come to CRP for rehabilitation service might be different to those who access services from other hospitals or NGOs, and those who do not seek any rehabilitation service at all. However, the CRP is a specialised treatment and rehabilitation centre for people with physical disabilities in Bangladesh, thus receives clients from all parts of Bangladesh. Furthermore, respondents were purposefully chosen to ensure that they represented people with different degrees of disability, different socio economic groups, rural and urban residence, both genders and different geographic areas of Bangladesh.

## Conclusions

The purpose of this study was to identify disability indicators, suitable for adults in Bangladesh, for a LDS to be used in community settings to measure impact of any LD rehabilitation programme on functioning/disability. Inclusion of items from all aspects of occupational areas (ADL, work, and leisure) and mobility activities and involvement of adults with LDs in identifying disability indicators are expected to enable the proposed LDS to accurately measure disabilities in real life situations.

Though this 70 item LDS is expected to have excellent content validity, its psychometric properties are subject to evaluation in order to accept or reject it as a valid and reliable measure of LDs among adults in Bangladesh.

## Additional files

Additional file 1: Table S1. Topic guide for the semi-structured interviews. **Table S2.** First round cognitive interview: example questions. **Table S3.** Second round cognitive interview: example questions. (DOCX 17 kb)

Additional file 2. The Locomotor Disability Scale (LDS). (DOCX 41 kb)

## Abbreviations

ADL: Activities of daily living
AOTA: American occupational therapy association
BDT: Bangladeshi taka
BI: Barthel index
CRP: Centre for the rehabilitation of the paralysed
FIM: Functional independence measure
HiMAT: High level mobility assessment tool
ICF: International classification of functioning, disability and health
LD: Locomotor disability
LDS: Locomotor disability scale
NGO: Non-government organization
PLDs: People with locomotor disabilities
PWDs: People/person with disabilities
RAP: Rehabilitation activities profile
RMI: Rivermead mobility index
SF-36: Short-form 36 health status questionnaire
USD: United States dollar
WHODAS 2.0: World Health Organization disability assessment schedule 2.0
