# Supplementary material for: Developing the content of a locomotor disability scale for adults in Bangladesh: a qualitative study
Source: Arch Physiother. 2017 Jun 6;7:7. doi: 10.1186/s40945-017-0035-7 (PMC5759907; doi:10.1186/s40945-017-0035-7)
Supplement: Supplementary file 1 — Topic guide for the semi-structured interviews. Table S2. First round cognitive interview: example questions. Table S3. Second round cognitive interview: example questions. (DOCX 17 kb) [file 40945_2017_35_MOESM1_ESM.docx]

**Additional file 1: Table S1 Topic guide for the semi-structured interviews**

| **Topics** | **Probe questions** |
| --- | --- |
| Mobility impairment | Do you have any permanent or chronic impairment which is affecting your mobility?  Would you mind describing it for me? |
| Impact on mobility | Does it affect your basic body movements-changing and maintaining body positions- and locomotion?  How does it affect your body movements?  How does it affect your walking and moving inside and outside your home?  How does it affect your travelling using available means of transports? |
| Impact on occupational performance areas: | |
| Activities of daily living | Does it affect your performance in self-care activities?  What are these self-care activities? [list all activities mentioned and ask the following question for each of the activities mentioned]  How does it affect your performance in [name of the activity]? |
| Work activities  Domestic/household activities  Occupation/job | Does it affect your participation in domestic/household activities?  What are these domestic activities? [list all activities mentioned and ask the following question for each of the activities mentioned]  How does it affect your participation in [name of the activity]?  Does it affect your participation in your job/occupation related activities?  What are these activities? [list all activities mentioned and ask the following question for each of the activities mentioned]  How does it affect your participation in [name of the activity]? |
| Leisure activities | Does it affect your participation in leisure activities?  What are these leisure activities? [list all activities mentioned and ask the following question for each of the activities mentioned]  How does it affect your participation in [name of the activity]? |

**Additional file 1: Table S2 First round cognitive interview: example questions**

| Currently, how much difficulty do you have in…? | **None** | **Mild** | **Moderate** | **Severe** | **Extreme** | | **N/A** |
| --- | --- | --- | --- | --- | --- | --- | --- |
| Taking a bath or shower | 0 | 1 | 2 | 3 | 4 | | 9 |
| **Interviewers to complete:** Did the respondent…. | | | | | | | |
| …need you to repeat any part of the question? | | | | Yes | | No | |
| …have any difficulty using the response categories? | | | | Yes | | No | |
| …ask for any clarification? | | | | Yes | | No | |
| **Ask.** Could you please tell me, why did you say that you have (none/mild/moderate/severe/extreme) difficulty in this activity?  **Ask.** What did you understand by ‘taking a bath or shower’?  **Ask.** When you scored your level of difficulty in ‘taking a bath or shower’, what were the specific tasks involved in this activity that you thought about? | | | | | | | |

**Additional file 1: Table S3 Second round cognitive interview: example questions**

| Currently, how much difficulty do you have in…? | **None** | **Mild** | **Moderate** | **Severe** | **Extreme** | | **N/A** |
| --- | --- | --- | --- | --- | --- | --- | --- |
| Taking a bath or shower | 0 | 1 | 2 | 3 | 4 | | 9 |
| **Tel:** This activity includes accessing the designated place, such as bathroom, obtaining and applying water, soap and other substances to the whole body in order to clean oneself, drying the whole body using a towel or other means. | | | | | | | |
| **Interviewer to complete:** Did the respondent…. | | | | | | | |
| …need you to repeat any part of the question? | | | | Yes | | No | |
| …have any difficulty using the response categories? | | | | Yes | | No | |
| …ask for any clarification? | | | | Yes | | No | |
| **Ask.** Could you please tell me, why did you say that you have (none/mild/moderate/severe/extreme) difficulty in this activity?  **Ask.** What did you understand by ‘taking a bath or shower’?  **Ask.** When you scored your level of difficulty in ‘taking a bath or shower’, what were the specific tasks involved in this activity that you thought about? | | | | | | | |
