# Supplementary material for: Developing the content of a locomotor disability scale for adults in Bangladesh: a qualitative study
Source: Arch Physiother. 2017 Jun 6;7:7. doi: 10.1186/s40945-017-0035-7 (PMC5759907; doi:10.1186/s40945-017-0035-7)
Supplement: Supplementary file 2 — The Locomotor Disability Scale (LDS). (DOCX 41 kb) [file 40945_2017_35_MOESM2_ESM.docx]

| **The Locomotor Disability Scale (LDS)** |
| --- |

***Keep displayed*** *this flash card in front of the respondent throughout the interview.*

| **Response options** | **Score** |
| --- | --- |
| **No difficulty**  Can perform on your own without any difficulties or with negligible difficulties. | 0 |
| **Mild difficulty**  Can perform on your own but with mild difficulties. Need more time and effort than usual. No risk involved and do not need any assistance of others. | 1 |
| **Moderate difficulty**  Cannot perform on your own. Need mild physical assistance or supervision of one person. If perform alone then there is a risk/history of fall or a long time period and/or severe effort is required. | 2 |
| **Severe difficulty**  Cannot perform on your own. Need moderate physical assistance of one person. | 3 |
| **Extreme difficulty**  Cannot perform on your own. Need total or maximum physical assistance of others (often two persons). | 4 |
| **Not applicable**  Do not or cannot perform because of reasons not related with health condition. | 99 |

**Flash card:** Response options for the LDS (0, no difficulty, to 4, extreme difficulty)

| **Locomotor Disability Scale (LDS)**  **[Tell:** *Now I will ask you about difficulties you are currently experiencing in mobility, self-care, work and leisure activities.*  **Tell:** *Even if you have no difficulties, it is necessary that I ask all of the questions for completeness. Your replies to all of the questions are very important for this research.*  **Tell:** *If you use any assistive device/aids, tell me how much difficulty you experience currently in performing activities even after using those devices/aids.*  **Tell:** *If you do not or cannot perform an activity because of reasons not related with your health condition, we shall mark that activity as ‘not applicable’ for you.*  **Tell:** *If any question has two parts, rate the one most difficult to you.*  ***Keep displayed*** *the response options flash card throughout the interview.***]** |
| --- |

| **Sl. No** | **Activities** | **Levels of difficulty** | | | | | |
| --- | --- | --- | --- | --- | --- | --- | --- |
|  |  | **No (0)** | **Mild (1)** | **Moderate (2)** | **Severe (3)** | **Extreme (4)** | **N/A (99)** |
| **Mobility:** *At first I will ask you some questions to know the levels of difficulty you are currently experiencing in mobility.*  Currently, how much difficulty do you have in...? | | | | | | | |
|  | **Sitting up from lying down** on your bed? |  |  |  |  |  |  |
|  | **Lying down from sitting up** on your bed? |  |  |  |  |  |  |
|  | **Standing up from sitting on a chair** or bed side? |  |  |  |  |  |  |
|  | **Sitting down on a chair** or bedside from a standing position? |  |  |  |  |  |  |
|  | **Sitting down on floor** from a standing position or any other sitting position, such as on a chair, wheelchair or bed? |  |  |  |  |  |  |
|  | **Standing up/sitting up** on a wheelchair from a sitting position on floor? |  |  |  |  |  |  |
|  | **Getting into a squatting position** that is sitting on your feet without a seat, maybe necessary in toilets that are at floor level? |  |  |  |  |  |  |
|  | **Getting out of a squatting position** that is standing up from a squatting position? |  |  |  |  |  |  |
|  | **Bending down and sideways** that is **b**ending/tilting your back downwards or to the side, at the torso, such as in bowing or reaching down for an object? |  |  |  |  |  |  |
|  | **Lifting any object** weighing approximately two kilograms, such as a jug full of water or other everyday objects? |  |  |  |  |  |  |
|  | **Reaching for overhead objects** by shifting body weight on the toes |  |  |  |  |  |  |
|  | **Transferring oneself from a sitting position to another sitting position**, such as moving from a bed to a chair or wheelchair and vice versa |  |  |  |  |  |  |
|  | **Maintaining a lying position** for some time as required, such as remaining in a prone position on your bed? |  |  |  |  |  |  |
|  | **Maintaining a squatting position** for some time as required (maybe necessary in toilets that are at floor level)? |  |  |  |  |  |  |
|  | **Maintaining a sitting position on a chair** on a chair or bedside for some time as required, such as maintaining a sitting position on a chair while eating? |  |  |  |  |  |  |
|  | **Maintaining a sitting position on floor** for some time as required, such as maintaining a sitting position on floor while eating? |  |  |  |  |  |  |
|  | **Maintaining a standing position** for some time as required, such as when standing in a queue? |  |  |  |  |  |  |
|  | **Walking inside your home**, such as walking within or around rooms and hallways, between rooms or in the yard? |  |  |  |  |  |  |
|  | **Walking in the neighbourhood**, such as across your village or town, across open areas or down the road? |  |  |  |  |  |  |
|  | **Walking on different surfaces** including slope, uneven, slippery or moving surfaces, such as on grass, gravel, sand, mud, slippery road or on moving vehicle? |  |  |  |  |  |  |
|  | **Walking around obstacles** in ways required avoiding moving and immobile objects, people, animals or vehicles, such as walking around a market place, through traffic or other crowded areas? |  |  |  |  |  |  |
|  | **Crawling inside home** in order to move from one place to another, such as crawling within or around rooms and hallways, between rooms or in the yard? |  |  |  |  |  |  |
|  | **Crawling in the neighbourhood** in order to move from one place to another, such as across your village or town, across open areas or down the road? |  |  |  |  |  |  |
|  | **Rolling inside your home** in order to move from one place to another, such as within or around rooms and hallways, between rooms or in the yard? |  |  |  |  |  |  |
|  | **Rolling in the neighbourhood** in order to move from one place to another, such as across your village or town, across open areas or down the road? |  |  |  |  |  |  |
|  | **Climbing up and down two flights of stairs?** |  |  |  |  |  |  |
|  | **Walking in quick steps**, such as to catch a public bus about to leave? |  |  |  |  |  |  |
|  | **Jumping** that is moving up off the ground by bending and extending the legs to travel some distance when required, such as to avoid an obstacle? |  |  |  |  |  |  |
|  | **Moving around inside your home using a wheelchair**, such as moving within or around rooms and hallways, between rooms or in the yard? |  |  |  |  |  |  |
|  | **Moving in your neighbourhood using a wheelchair**, such as across your village or town, across open areas or down the road, using a wheelchair? |  |  |  |  |  |  |
|  | **Carrying objects** up to 5 kg, such as a shopping bag while walking or propelling a wheelchair? |  |  |  |  |  |  |
|  | **Getting into and out of own home?** |  |  |  |  |  |  |
|  | **Travelling by non-motorised vehicles**, such as riding on a rickshaw? |  |  |  |  |  |  |
|  | **Travelling by private motorised vehicles**, such as by own or hired taxi? |  |  |  |  |  |  |
|  | **Travelling by public transports**, such as by bus or train? |  |  |  |  |  |  |
|  | **Driving human-powered vehicles**, such as a bicycle or tricycle/rickshaw? |  |  |  |  |  |  |
|  | **Driving motorised vehicles**, such as a car or motorcycle? |  |  |  |  |  |  |
| **Occupational Performance Areas** | | | | | | | |
|  | | | | | | | |
| **Activities of Daily Living (ADL):** *Now I will ask you some questions to know the levels of difficulty you are currently experiencing to perform some of your ADL.*  Currently, how much difficulty do you have in...? | | | | | | | |
|  | **Washing your body parts?**  This activity includes accessing the designated place, such as wash-basin, obtaining and applying supplies, such as water, soap and other substances to body parts, such as hands, face, feet or hair in order to clean them when required, drying the body parts using a towel or other means. |  |  |  |  |  |  |
|  | **Taking a bath or shower?**  This activity includes accessing the designated place and obtaining and applying water, soap and other substances to the whole body in order to clean oneself and then drying whole body using a towel or other means. |  |  |  |  |  |  |
|  | **Grooming?**  This activity includes accessing and using necessary supplies in order to look after those parts of the body, such as skin, face, teeth, hair, nails and genitals, that require more than just washing and drying, such as shaving face, having a hair cut or applying lotion to body parts. |  |  |  |  |  |  |
|  | **Toileting?**  This activity includes accessing the toilet, carrying out the elimination of human waste (menstruation, urination and defecation), obtaining and using the supplies (water, tissue paper and soap) in order to clean oneself afterwards. |  |  |  |  |  |  |
|  | **Dressing?**  This activity includes collecting clothes, footwear, personal devices, such as prostheses, or orthoses, from storage area and putting on or taking off these and keeping them in the storage area. |  |  |  |  |  |  |
|  | **Feeding?**  This activity includes obtaining food, drinks and utensils from storage area, accessing the appropriate places, such as dining table/dining room, and then using hand and or appropriate utensils and tableware for eating and drinking. |  |  |  |  |  |  |
|  | **Maintaining own health?**  This activity includes responding to risks to health and to prevent ill-health, such as by seeking professional assistance, following medical and other health advice. |  |  |  |  |  |  |
|  | **Praying?**  This activity includes accessing prayer place, such as mosque, church or any other designated place and performing prayers. |  |  |  |  |  |  |
|  | **Attending Ceremonies?**  This activity includes attending social and cultural ceremonies, such as marriage or funerals ceremonies. |  |  |  |  |  |  |
|  | | | | | | | |
| **Productive Activities:** *Now I will ask you some questions to know the levels of difficulty you are currently experiencing to perform some of your household and work activities.*  Currently, how much difficulty do you have in...? | | | | | | | |
|  | **Shopping?**  This activity includes accessing a shop or market, buying goods for family and transporting these to home. |  |  |  |  |  |  |
|  | **Cooking?**  This activity includes accessing kitchen, getting together ingredients for cooking, cooking using appropriate kitchen utensils and appliances and storing food safely. |  |  |  |  |  |  |
|  | **Serving meals?**  This activity includes obtaining food and drinks items from a storage area, serving in an appropriate place, cleaning up and storing back items in the storing place. |  |  |  |  |  |  |
|  | **Cleaning cooking area & utensils?**  This activity includes cleaning up after cooking using appropriate supplies, such as washing dishes, pans and pots, and cleaning cooker, tables and floors around cooking area. |  |  |  |  |  |  |
|  | **Washing and drying clothes and garments?**  This activity includes washing clothes and garments by hand and hanging them out to dry in the air or washing and drying using a washing machine and dryer. |  |  |  |  |  |  |
|  | **Cleaning living area?**  This activity includes tidying, dusting, sweeping, swabbing and mopping floors, cleaning windows and walls, cleaning bathrooms and toilets, and cleaning household furnishings. |  |  |  |  |  |  |
|  | **Disposing of household garbage?**  This activity includes collecting trash and rubbish around the house, preparing garbage for disposal, using garbage disposal appliances and disposing them of in an appropriate place. |  |  |  |  |  |  |
|  | **Maintaining dwelling and furnishings?**  This activity includes repairing and taking care of dwelling by yourself or by employing people whichever is appropriate. |  |  |  |  |  |  |
|  | **Taking care of domestic/pet animals?**  This activity includes feeding, cleaning, grooming and exercising domestic animals and pets and watching over their health. |  |  |  |  |  |  |
|  | **Assisting household members with self-care?**  This activity includes helping other household members with eating, bathing and dressing, such as taking care of children or household members who are sick or have difficulties with self-care activities. |  |  |  |  |  |  |
|  | **Assisting household members in movement?**  This activity includes helping them in moving inside and outside the home, such as between rooms and in the neighbourhood. |  |  |  |  |  |  |
|  | **Assisting household members in health maintenance?**  This activity includes assisting them in seeking health care and following health advice, such as assisting them to visit clinics and to take required medications. |  |  |  |  |  |  |
|  | **Accessing public services?**  This activity includes accessing services, such as health, utility, bank, social security, food security etc. |  |  |  |  |  |  |
|  | **Seeking employment?**  This activity includes locating and choosing a job in a trade, profession or other form of employment, and performing the required tasks to get hired, such attending job interviews. |  |  |  |  |  |  |
|  | **Maintaining an occupation?**  This activity include performing job-related tasks to keep an occupation, trade, profession or other form of employment, and obtaining promotion and other advancements in employment. |  |  |  |  |  |  |
|  | **Agricultural activities?**  This activity includes growing and selling crops by own self or by hiring people. |  |  |  |  |  |  |
|  | **Doing voluntary social work?**  This activity includes performing formal or informal unpaid social work. |  |  |  |  |  |  |
|  | | | | | | | |
| **Leisure Activities:** *Now I will ask you some questions to know the levels of difficulties you are experiencing in performing some of your leisure activities.*  Currently, how much difficulty do you have in...? | | | | | | | |
|  | **Socialising?**  This activity includes engaging in informal or casual gatherings with others, such as visiting friends or relatives or meeting informally in public places. |  |  |  |  |  |  |
|  | **Playing in-door games?**  This activity includes playing competitive in-door games, performed alone or in a group, such as playing cards, chess etc. |  |  |  |  |  |  |
|  | **Playing out-door games?**  This activity includes playing competitive out-door games or athletic events, performed alone or in a group, such as football, cricket, running etc. |  |  |  |  |  |  |
|  | **Attending arts, cultural and sports events?**  This includes going to theatre, cinema, museum, art gallery or stadium. |  |  |  |  |  |  |
|  | **Travelling for pleasure?**  This includes visiting a place for pleasure and interest, usually on holidays. |  |  |  |  |  |  |
|  | **Gardening?**  This activity includes working in a garden for pleasure, growing and taking care of plants. |  |  |  |  |  |  |
|  | **Watching television?**  This includes accessing the television, switching it on and off and selecting programmes of interest to watch. |  |  |  |  |  |  |

**Instructions on computing total LDS score and handling missing values:**

The score can be calculated by the application of latent variable models or for simplicity when appropriate software is not available by summing the items and dividing by 70.

When latent variable models are used to derive the total score, principled methods for missing data (including the response option ‘N/A’) handling such as Multiple Imputation (MI) and Full Information Maximum Likelihood (FIML) can be used to deal with missing values. If the simple sum of the items is employed, replacing missing values by the mean of the observed values is an option, but may result to biased (shrink) standard errors.
